# People with Suspected COVID-19 Symptoms Were More Likely Depressed and Had Lower Health-Related Quality of Life: The Potential Benefit of Health Literacy

**DOI:** 10.3390/jcm9040965

**Published:** 2020-03-31

**Authors:** Hoang C. Nguyen, Minh H. Nguyen, Binh N. Do, Cuong Q. Tran, Thao T. P. Nguyen, Khue M. Pham, Linh V. Pham, Khanh V. Tran, Trang T. Duong, Tien V. Tran, Thai H. Duong, Tham T. Nguyen, Quyen H. Nguyen, Thanh M. Hoang, Kien T. Nguyen, Thu T. M. Pham, Shwu-Huey Yang, Jane C.-J. Chao, Tuyen Van Duong

**Affiliations:** 1 Director Office, Thai Nguyen National Hospital, Thai Nguyen City 241-24, Vietnam; nguyenconghoang@tnmc.edu.vn (H.C.N.); dhthaivn@gmail.com (T.H.D.); 2 President Office, Thai Nguyen University of Medicine and Pharmacy, Thai Nguyen City 241-17, Vietnam; 3 International Master/Ph.D. Program in Medicine, Taipei Medical University, Taipei 110-31, Taiwan; drminh.ttytlc@gmail.com (M.H.N.); phamminhthu.ytcc@gmail.com (T.T.M.P.); 4 Department of Infectious Diseases, Military Medical University, Hanoi 121-08, Vietnam; nhubinh.do@vmmu.edu.vn (B.N.D.); tientv@vmmu.edu.vn (T.V.T.); 5 Division of Military Science, Military Medical Hospital 103, Hanoi 121-08, Vietnam; 6 Department of Anesthesiology, Thu Duc District Hospital, Ho Chi Minh City 713-11, Vietnam; quoccuong.mph@gmail.com (C.Q.T.); huuquyenhm@gmail.com (Q.H.N.); hoangminhthanhbvct@gmail.com (T.M.H.); 7 Director Office, Thu Duc District Health Center, Ho Chi Minh City 713-10, Vietnam; 8 Health Management Training Institute, Hue University of Medicine and Pharmacy, Thua Thien Hue 491-20, Vietnam; ntpthao.hmti@huemed-univ.edu.vn; 9 School of Health Policy, Planning and Financing, Corvinus University of Budapest, Budapest 1093, Hungary; 10 Faculty of Public Health, Hai Phong University of Medicine and Pharmacy, Hai Phong 042-12, Vietnam; pmkhue@hpmu.edu.vn (K.M.P.); nttham@hpmu.edu.vn (T.T.N.); 11 President Office, Hai Phong University of Medicine and Pharmacy, Hai Phong 042-12, Vietnam; 12 Department of Pulmonary & Cardiovascular Diseases, Hai Phong University of Medicine and Pharmacy Hospital, Hai Phong 042-12, Vietnam; pvlinh@hpmu.edu.vn; 13 Director Office, Hai Phong University of Medicine and Pharmacy Hospital, Hai Phong 042-12, Vietnam; 14 Director Office, Hospital District 2; Ho Chi Minh City 711-13, Vietnam; tvkhanh.q2@tphcm.gov.vn; 15 Nursing Office, Tan Phu District Hospital; Ho Chi Minh City 720-16, Vietnam; duongtrang7273@gmail.com; 16 Director Office, Military Medical Hospital 103, Hanoi 121-08, Vietnam; 17 Department of Internal Medicine, Thai Nguyen University of Medicine and Pharmacy, Thai Nguyen City 241-17, Vietnam; 18 Department of Health Education, Faculty of Social Sciences, Behavior and Health Education, Hanoi University of Public Health, Hanoi 119-10, Vietnam; ntk1@huph.edu.vn; 19 School of Nutrition and Health Sciences, Taipei Medical University, Taipei 110-31, Taiwan; sherry@tmu.edu.tw (S.-H.Y.); chenjui@tmu.edu.tw (J.C.-J.C.); 20 Research Center of Geriatric Nutrition, Taipei Medical University, Taipei 110-31, Taiwan; 21 Nutrition Research Center, Taipei Medical University Hospital, Taipei 110-31, Taiwan; 22 Master Program in Global Health and Development, College of Public Health, Taipei Medical University, Taipei 110-31, Taiwan

**Keywords:** coronavirus, COVID-19, epidemic, suspected COVID-19 symptoms, comorbidity, healthy eating, physical activity, health literacy, depression, health-related quality of life

## Abstract

The coronavirus disease 2019 (COVID-19) epidemic affects people’s health and health-related quality of life (HRQoL), especially in those who have suspected COVID-19 symptoms (S-COVID-19-S). We examined the effect of modifications of health literacy (HL) on depression and HRQoL. A cross-sectional study was conducted from 14 February to 2 March 2020. 3947 participants were recruited from outpatient departments of nine hospitals and health centers across Vietnam. The interviews were conducted using printed questionnaires including participants’ characteristics, clinical parameters, health behaviors, HL, depression, and HRQoL. People with S-COVID-19-S had a higher depression likelihood (OR, 2.88; *p* < 0.001), lower HRQoL-score (B, −7.92; *p* < 0.001). In comparison to people without S-COVID-19-S and low HL, those with S-COVID-19-S and low HL had 9.70 times higher depression likelihood (*p* < 0.001), 20.62 lower HRQoL-score (*p* < 0.001), for the people without S-COVID-19-S, 1 score increment of HL resulted in 5% lower depression likelihood (*p* < 0.001) and 0.45 higher HRQoL-score (*p* < 0.001), while for those people with S-COVID-19-S, 1 score increment of HL resulted in a 4% lower depression likelihood (*p* = 0.004) and 0.43 higher HRQoL-score (*p* < 0.001). People with S-COVID-19-S had a higher depression likelihood and lower HRQoL than those without. HL shows a protective effect on depression and HRQoL during the epidemic.

## 1. Backgrounds

The outbreak of novel coronavirus disease (COVID-19) was first reported in December 2019 in Wuhan, China [1]. COVID-19 has been spreading to a growing number of countries and is recognized as a global health concern that has set global public health institutions on high alert [2,3,4]. The World Health Organization (WHO) declared the COVID-19 emergency to be the sixth public health emergency of international concern (PHEIC) on 30 January 2020 [5]. According to the report on 24 March 2020, the total number of people diagnosed with COVID-19 was 372,757 cases, with 16,231 deaths in 170 countries/regions; among these, 123 cases have been reported in Vietnam [6,7]. COVID-19 was officially declared a pandemic by WHO on 11 March 2020 [8]. Treatments and vaccines for COVID-19 are currently in development. Therefore, prevention and supportive care are highly recommended [7,9,10,11,12,13], especially in countries with weaker healthcare systems, as suggested by WHO director-general Tedros Adhanom Ghebreyesus [14]. Vitally, intense surveillance is emphasized to prevent the sustained transmission in new locations [15]. The public health response to prevent the spread of COVID-19 has been initiated at different levels in all countries affected [16].

The COVID-19 pandemic causes panic and mental health problems for the public [17,18], as experienced previously with the Middle-East respiratory syndrome coronavirus (MERS-CoV) [19,20]. Additionally, myths and misinformation about this epidemic, travel bans and executive orders to quarantine travelers might affect the public’s psychological health [17,21,22]. This may influence people’s health and quality of life. Health literacy (HL) is defined as the ability to find, understand, appraise and apply health-related information that could help healthcare systems and individuals achieve a better quality of care, lifestyle, disease management, treatment decision, and health outcomes [23,24]. Health literacy is recognized as a key factor in reducing health inequities and improving health and well-being [25,26]. Adequate HL enables health-friendly environments, efficient health policies implementation, effective health promotional efforts, better self-care, healthcare outcomes, and lower expenditures [27].

Vietnam has a long border and several bilateral communications with China [28]. This puts Vietnam at high risk for the COVID-19 pandemic. Vietnam also has the lowest health literacy score among studied Asian countries [29,30]. Health literacy is considered to be an essential element of public health practice to generate effective interventions and maximize health outcomes [31,32]. Improving health literacy is a strategic approach to preventing and controlling diseases [33]. Therefore, health professionals need to understand patients’ health literacy before delivering interventions or education [34], in order to improve health-related quality of life [35,36].

People with health problems and who need to visit clinics are vulnerable populations. The COVID-19 pandemic causes panic and anxiety, which further exaggerates their health outcomes, especially for those who present at outpatient departments (OPD) with suspected COVID-19 symptoms (S-COVID-19-S). From a public health perspective, it is crucial to find the protective factors that benefit daily health-related behaviors, mental health, and quality of life. We hypothesized that people with S-COVID-19-S are more likely to have depression and lower health-related quality of life (HRQoL), while those with higher HL have better mental health and HRQoL during the COVID-19 pandemic. Therefore, we aimed to investigate the association of S-COVID-19-S with depression and HRQoL, and the effect modification of health literacy on the associations among people who visited OPDs of 9 hospitals and health centers in Vietnam.

## 2. Research Methods

### 2.1. Study Design and Settings

A cross-sectional study was conducted from 14 February to 2 March 2020. Participants were recruited from outpatient departments (OPD) of six hospitals and three health centers across Vietnam, including three hospitals and one health center in the North, one health center in the Center, and three hospitals and one health center in the South.

### 2.2. Sampling and Sample Size

People who visited the OPD in hospitals and health centers were invited to the survey. Studied participants were aged 18 to 85 years, understood the Vietnamese language, and were not in any emergency condition. Patients with a diagnosis of psychotic disorder, dementia, or blindness were excluded, to avoid interaction of health literacy measurements [37].

A total sample of 4029 participants was interviewed, including 500 from Thai Nguyen National Hospital in Thai Nguyen Province, 530 from the Military Medical Hospital 103 in Hanoi, 502 from Hai Phong University of Medicine and Pharmacy Hospital and 497 from Kien Thuy District Health Center in Haiphong city, 500 from Trieu Phong District Health Center, Thua Thien Hue province, 500 from Thu Duc Hospital, 250 from Tan Phu District Hospital, and 250 from Hospital District 2, and 500 from Thu Duc District Health Center in Ho Chi Minh city. After cleaning the data (82 individuals aged less than 18 or above 85 years, or with incomplete interviews, were excluded), the sample of 3947 participants was analyzed.

### 2.3. Research Instruments and Assessments

#### 2.3.1. Social Demographics and Clinical Indicators

Participants were asked about the reason to visit the OPD, and screened for suspected COVID-19 symptoms (S-COVID-19-S) [38], e.g., common symptoms (fever, cough, dyspnea), and less common symptoms (myalgia, fatigue, sputum production, confusion, headache, sore throat, rhinorrhea, chest pain, hemoptysis, diarrhea, and nausea/vomiting). People presenting at OPD with any of those symptoms were classified as having S-COVID-19-S. They were also asked about age (years), gender (women vs. men), marital status (never married vs. ever married), education (elementary/illiterate, junior high school, senior high school, college/university or above), occupation (employed, own business, others), ability to pay for medication (very difficult to very easy), social status (people were asked to place themselves into the society at three levels from low, middle to high), body height (cm), weight (kg), and comorbidity. Body mass index (BMI, kg/m^2^) was calculated.

#### 2.3.2. Health-Related Behaviors

Participants were asked about the current smoking status (yes vs. no), drinking status (yes vs. no), eating behavior (less healthy or unchanged vs. healthier during the outbreak). The physical activity was assessed using the short version of the International Physical Activity Questionnaire (IPAQ) with seven items was used to evaluate the physical activity level, which was widely used [39]. Patients were asked about their time spent (days per week, and minutes per day) over the last 7 days, on different levels of physical intensity (vigorous, moderate, walking, and sitting). The IPAQ was validated and used in the Vietnamese context [40,41]. The overall physical activity was scored using a metabolic equivalent task scored in minutes per week (named as MET-min/week) [42]. The MET-min/week was calculated as the sum of minutes spent on activities at different levels of vigorous, moderate, walking, and sitting over the last seven days multiplied by 8.0, 4.0, and 3.3, 1.0, respectively [39].

#### 2.3.3. Health Literacy

Health literacy (HL) was measured using the 12-item short form of the health literacy questionnaire (HLS-SF12), which has been validated in general populations in Asia [30], and in Vietnam [43]. Participants rated their perceived difficulty of each item on 4-point Likert scales (1 = very difficult, 2 = difficult, 3 = easy, and 4 = very easy). The HL index was standardized to unified metrics from 0 to 50 using the formula;

Index = (Mean − 1)*(50/3) (1)

where *Index* is the specific index calculated, *Mean* is the mean of all participating items for each individual, *1* is the minimum possible value of the mean (leading to a minimum value of the index of 0), *3* is the range of the mean, and *50* is the chosen maximum value of the new metric. Thus, a higher HL index score represents a better HL level [44].

#### 2.3.4. Depression

Depression was assessed using the patient health questionnaire (PHQ-9) [45]. The PHQ-9 is in use in the Vietnam context [40,46]. Participants rated each item on a 4-point Likert scale from 0 (not at all) to 3 (almost every day) over the last 2 weeks. Total PHQ-9 score ranges from 0–27. Scores ≥ 10 are classified as depression [45,47,48].

#### 2.3.5. Health-Related Quality of Life

Health-related quality of life (HRQoL) was assessed using the 36-Item Short Form Survey (SF-36) which was developed at RAND as part of the Medical Outcomes Study [49]. This was also used in Vietnamese Americans [50]. Scoring algorithms are given in detail in the user manual [51,52]. The possible calculated scores range from 0 to 100, with a higher score representing a better HRQoL.

### 2.4. Data Collection Procedure

Research assistants (doctors, nurses, and medical students) received a 4 h training session on data collection by two senior researchers at each hospital or health center. The infection control training was also provided, e.g., using the mask, washing hands, positioning according to guidelines of the Centers for Disease Control and Prevention (CDC) [53], and World Health Organization (WHO) [54].

All qualified patients were invited to participate in the survey. Research assistants contacted and asked for voluntary participation from patients and relatives who visit the OPD. The consent form was obtained before administering the survey. The interviews were then conducted at the OPD. It takes about 30 min to complete the questionnaire. Finally, all the data was cleaned, keyed-in and analyzed confidentially by researchers.

### 2.5. Ethical Considerations

The study protocol was approved by each participating hospital, and the Institutional Ethical Review Committee of Hanoi School of Public Health, Vietnam (IRB No. 029/2020/YTCC-HD3).

### 2.6. Data Analysis

Firstly, descriptive analysis was used to explore the distribution of different variables. The chi-squared test and one-way ANOVA test were used, respectively. Secondly, we used bivariate and multivariate analyses to investigate the determinants of depression and HRQoL. The factors associated with depression or HRQoL at *p* < 0.20 in the bivariate model were selected into the multivariate model [55]. Finally, the interaction analysis was conducted to examine the potential benefit of health literacy on improving depression and HRQoL. The significance level was set at a *p*-value < 0.05. All statistical analyses were performed using the IBM SPSS Version 20.0 (IBM Corp, Armonk, NY, USA) [56].

## 3. Results

### 3.1. Participants’ Characteristics

The mean age of the study population was 44.4 ± 17.0 years, with 23.5% of them aged 60 years or above. Out of all participants, 35.1% had presented at the outpatient department with suspected COVID-19 symptoms (S-COVID-19-S), 44.3% were men, and 7.4% were depressed. The mean scores of HL index and HRQoL were 29.9 ± 7.7 and 69.6 ± 17.5, respectively. The prevalence of depression was significantly higher in people with S-COVID-19-S, and varied by different groups of age, education, ability to pay for medication, social status, comorbidity, eating behavior, physical activity, and had lower HL score (*p* < 0.05; Table 1). The HRQoL score was significantly lower in the people with S-COVID-19-S, and varied by different categories of age, gender, marital status, education, occupation, ability to pay for medication, social status, comorbidity, and physical activity (*p* < 0.001; Table 1). Additionally, the HRQoL score was significantly lower in the people with depression (*p* < 0.001; Table 1).

### 3.2. Depression

In the bivariate analysis, the odds of depression were significantly higher in people with S-COVID-19-S, aged 60 years or above, and with comorbidity (*p* < 0.05; Table 2). The odds of depression were significantly lower in people with higher education attainment, who found it very or fairly easy to pay for medication, with middle or high social status, who ate healthier, had more physical activity, and had a higher HL as compared to their counterparts (*p* < 0.05; Table 2). In multivariate analysis, as compared to the people without S-COVID-19-S, those people with S-COVID-19-S had a higher likelihood of depression (odds ratio, OR, 2.88; 95% confidence interval, 95%CI, 2.18, 3.80, *p* < 0.001). People with college/university or above educational attainment had higher odds of depression as compared to those people with elementary school/illiterate attainment (OR, 2.12; 95%CI, 1.34, 3.35, *p* = 0.001). In comparison to the people who had a low social status, ate less healthy or unchanged, had less physical activity (tertile 1), those people with middle or high social status (OR, 0.45; 95%CI, 0.33, 0.63, *p* < 0.001), ate healthier (OR, 0.59; 95%CI, 0.42, 0.83, *p* = 0.003), had more physical activity (OR, 0.59; 95%CI, 0.42, 0.82, *p* = 0.001 for tertile 2; OR, 0.56; 95%CI, 0.40, 0.78, *p* = 0.001 for tertile 3) had lower odds of depression, respectively (Table 2). People with higher HL score had lower odds of depression (OR, 0.91; 95%CI, 0.90, 0.93, *p* < 0.001; Table 2).

### 3.3. Health-Related Quality of Life

In the bivariate analysis, the HRQoL score was significantly lower in people with S-COVID-19-S, older age, ever married, had own business or others, with comorbidity (*p* < 0.05; Table 2). The HRQoL score was significantly higher in men and in people with higher education attainment, with better ability to pay for medication, with middle or high social status, who did more physical activity, and who had higher HL as compared to their counterparts (*p* < 0.05; Table 2). In multivariate analysis, the HRQoL score was significantly lower in the people with S-COVID-19-S (regression coefficient, B, −7.92; 95% confidence interval, 95%CI, −8.95, −6.89, *p* < 0.001), aged 60 years or older (B, −3.60; 95%CI, −5.13, −2.08, *p* < 0.001), ever married (B, −2.82; 95%CI, −4.12, −1.51, *p* < 0.001), with comorbidity (B, −2.81; 95%CI, −4.18, −1.45, *p* < 0.001; Table 2). The HRQoL score was significantly higher in men (B, 1.89; 95%CI, 0.82, 2.95, *p* = 0.001), and in people with higher education attainment (B, 6.82; 95%CI, 4.85, 8.78, *p* < 0.001 for junior high school; B, 6.70; 95%CI, 4.71, 8.69, *p* < 0.001 for senior high school; and B, 4.70; 95%CI, 2.66, 6.75, *p* < 0.001 for college/university or higher), in people with their own business (B, 2.25; 95%CI, 0.73, 3.77, *p* = 0.004), in people with better ability to pay for medication (B, 2.75; 95%CI, 1.74, 3.76, *p* < 0.001), in people with middle or high social status (B, 4.62; 95%CI, 3.09, 6.15, *p* < 0.001), in people who did not drink (B, 1.74; 95%CI, 0.61, 2.87, *p* = 0.003), in those who did more physical activity (B, 2.72; 95%CI, 1.52, 3.92, *p* < 0.001 for the 3rd tertile), and in people with higher HL (B, 0.59; 95%CI, 0.52, 0.66, *p* < 0.001; Table 2).

### 3.4. Effect Modification of Health Literacy

The results of interaction analysis showed that as compared to people without S-COVID-19-S and low HL, those people with S-COVID-19-S and low HL had a 9.70 times higher likelihood of depression (OR, 9.70; 95%CI, 4.02, 23.41, *p* < 0.001) and a 20.62 lower HRQoL score (B, −20.62; 95%CI, −24.63, −16.62, *p* < 0.001); for people without S-COVID-19-S, 1 score increment of HL resulted in 5% lower likelihood of depression (OR, 0.95; 95%CI, 0.93, 0.97, *p* < 0.001) and 0.45 higher HRQoL score (B, 0.45; 95%CI, 0.37, 0.54, *p* < 0.001), while for those people with S-COVID-19-S, 1 score increment of HL had 4% lower likelihood of depression (OR, 0.96; 95%CI, 0.93, 0.99, *p* = 0.004) and 0.43 higher HRQoL score (B, 0.43; 95%CI, 0.30, 0.57, *p* < 0.001; Table 3). Since it is suspected that people with low HL, presenting less common symptoms only, may not suspect COVID-19 infection, we conducted a sensitivity analysis for the sample with less common symptoms excluded (*n* = 700). The results showed that the effect modification of HL on depression and HRQoL remained significant (Table S1).

## 4. Discussion

The most important finding of the current study was that people with S-COVID-19-S had a higher likelihood of depression and lower HRQoL. Fortunately, those people having S-COVID-19-S and with higher HL had a low occurrence of depression and better HRQoL compared to those people with lower HL. Health literacy was found to be a protective factor for improving depression and HRQoL during the COVID-19 epidemic, especially for those who have S-COVID-19-S. To improve health literacy and control the disease and its consequences during the outbreak, governments must firstly recognize COVID-19 as an emergency public health concern [5], and find the balance between public health and civil liberties [57], as well as cultural sensitivity [58]. The government needs to provide the public with updated, timely, accurate, transparent, brief, simple information and knowledge regarding the epidemic, pathogenicity, and transmissibility which help with better controlling the disease [58,59]. In Vietnam, the leaders of the government have been leading the government in actions against COVID-19 directly [60]. The healthcare and public health communities and global and domestic public health preparedness capacity buildings need to be sustained and strengthened in order to effectively respond to the epidemic [61,62]. The ministry of health of Vietnam has been directing all healthcare institutions at all levels and collaborating with other sectors to communicate with the public, in order to prevent and control the disease [7]. These messages apply for every government, especially in Vietnam, a country with the lowest HL level among all studied Asian countries [29,30]. The main reason for the low HL level may be linked with accessibility of reliable information. Therefore, it is critical for the government and the ministry of health to provide accessible platforms with official and reliable information regarding the COVID-19 pandemic. In addition, it is suggested that people should enhance their life-long learning behaviors (e.g., watching health-related television programs, reading official websites) to improve individual health literacy [63]. The above may further contribute to preventing and controlling infectious diseases [64,65].

We also found that older people had a lower HRQoL score compared to the younger age groups. This was consistent with previous studies in Vietnam [66,67,68]. Furthermore, men had a better HRQoL score than women did, which was found in the current study and in previous studies [68,69]. People with comorbidities had a lower HRQoL score than those people without, which was in the line with previous studies [68,70,71]. In respiratory patients, those who suffer from at least one chronic condition were significantly associated with decreased HRQoL [72].

The present study shows that people who have attained college/university level of education or above had a higher prevalence of depression. Members of this group usually have jobs and have to work every day, while their children’s schools have postponed the new semester [73]. Going to work and/or taking care of children during the pandemic creates a burden of stress which further affects their HRQoL, as found in the current study. Therefore, people with education at college/university level or above had slightly lower HRQoL as compared to those with high school levels of education [71]. Nevertheless HRQoL was still higher in people with higher levels of educational attainment in the current study and in the previous one [68]. Furthermore, people who had ever married had lower HRQoL. Logically, people who had their own businesses had better HRQoL than those who employed. In addition, participants with middle or high social status had a lower likelihood of depression and better HRQoL. People with higher social status are likely wealthier. The present study demonstrates that people with a better ability to pay for medication had better HRQoL. Similarly, previous studies have shown that higher income is positively associated with higher HRQoL in the general population [68,71].

Physical activity was positively associated with HRQoL, whereby people with more physical activity had better HRQoL [68]. Physical activity was also summarized as a protective factor of depression [74], and exercise is suggested to be an effective treatment for depression [75]. In addition, people who did not drink had better HRQoL than those who drank alcohol. The association between alcohol consumption and reduced HRQoL has been found in previous studies [76,77,78]. Healthy eating was associated with a lower likelihood of depression in the current study. Previous studies have elucidated that higher diet quality is associated with a lower risk of depression [79,80]. Dietary intake has been recommended for prevention of depression [81,82]. Therefore, healthy lifestyles are highly recommended to preventing depression and improving the quality of life during the COVID-19 pandemic.

There are limitations in the current study. Firstly, the study was conducted during the global COVID-19 outbreak, and both interviewers and the studied participants were vulnerable to coronavirus infection. This requires great effort on the parts of institutions, researchers, and assistants in order to strictly follow the safety guidelines during data collection. Fortunately, there no new cases were detected during the data collection period [7]. Secondly, causal relationships cannot be determined from this cross-sectional study. The sample size was adequate for exploring the associations and interactions that provide substantial evidence and directions for future studies and public health interventions for controlling the disease.

## 5. Conclusions

During the global COVID-19 pandemic, people with S-COVID-19-S were more likely to have depression and low HRQoL. Meanwhile, higher HL was associated with a lower depression likelihood and higher HRQoL. Importantly, a potential benefit of HL was found in that it can help to protect the mental health and HRQoL of people with S-COVID-19-S during the pandemic. Further studies are required to explore the benefits of HL in healthcare providers and medical students. Efforts of individuals and governments to improve health literacy can significantly contribute to COVID-19 prevention and control.

## Figures and Tables

**Table 1:** Participants’ characteristics, health literacy index score, depression and health-related quality of life of participants.

	Total (*n* = 3947)	PHQ < 10 (*n* = 3653)	PHQ ≥ 10 (*n* = 294)		HRQoL	
	Frequency (%)	Frequency (%)	Frequency (%)	*p* ^1^	Mean ± SD	*p* ^2^
Reasons to visit OPD *				<0.001		<0.001
Without S-COVID-19-S	2560 (64.9)	2455 (67.2)	105 (35.7)		73.6 ± 15.2	
With S-COVID-19-S	1387 (35.1)	1198 (32.8)	189 (64.3)		62.1 ± 18.8	
Age, year				<0.001		<0.001
18–39	1788 (45.3)	1691 (46.3)	97 (33.0)		74.3 ± 15.6	
40–59	1231 (31.2)	1158 (31.7)	73 (24.8)		69.6 ± 17.1	
≥60	928 (23.5)	804 (22.0)	124 (42.2)		60.6 ± 17.9	
Gender				0.171		<0.001
Women	2197 (55.7)	2022 (55.4)	175 (59.5)		68.3 ± 17.7	
Men	1747 (44.3)	1628 (44.6)	119 (40.5)		71.2 ± 17.1	
Marital status				0.957		<0.001
Never married	865 (22.0)	800 (22.0)	65 (22.1)		75.3 ± 15.5	
Ever married	3070 (78.0)	2841 (78.0)	229 (77.9)		67.9 ± 17.6	
Education				<0.001		<0.001
Elementary school or Illiterate	347 (8.8)	294 (8.1)	53 (18.0)		55.1 ± 19.7	
Junior high school	869 (22.1)	820 (22.5)	49 (16.7)		68.3 ± 16.4	
Senior high school	1083 (27.5)	1021 (28.0)	62 (21.1)		72.3 ± 15.5	
College/university or above	1639 (41.6)	1509 (41.4)	130 (44.2)		71.5 ± 17.2	
Occupation				0.108		<0.001
Employed	753 (19.2)	700 (19.3)	53 (18.1)		72.6 ± 17.9	
Own business	1402 (35.7)	1311 (36.1)	91 (31.1)		69.7 ± 17.2	
Others	1770 (45.1)	1621 (44.6)	149 (50.9)		68.1 ± 17.3	
Ability to pay for medication				<0.001		<0.001
Very or fairly difficult	1764 (44.7)	1589 (43.5)	175 (59.5)		65.1 ± 17.8	
Very or fairly easy	2182 (55.3)	2063 (56.5)	119 (40.5)		73.2 ± 16.3	
Social status				<0.001		<0.001
Low	482 (12.2)	396 (10.8)	86 (29.3)		59.5 ± 18.3	
Middle or high	3464 (87.8)	3256 (89.2)	208 (70.7)		71.0 ± 16.9	
BMI, kg/m^2^				0.085		0.241
Normal weight (BMI < 25.0)	3514 (89.1)	3244 (88.9)	270 (92.2)		69.5 ± 17.7	
Overweight/obese (BMI ≥ 25.0)	428 (10.9)	405 (11.1)	23 (7.8)		70.5 ± 15.6	
Comorbidity				0.034		<0.001
None	3309 (84.4)	3076 (84.8)	233 (80.1)		70.8 ± 16.8	
One or more	611 (15.6)	553 (15.2)	58 (19.9)		63.3 ± 19.4	
Smoking				0.583		0.662
Smoke	471 (12.0)	433 (11.9)	38 (13.0)		69.9 ± 18.4	
No smoke	3465 (88.0)	3210 (88.1)	255 (87.0)		69.6 ± 17.3	
Drinking alcohol				0.379		0.080
Drink	1257 (32.1)	1170 (32.3)	87 (29.8)		70.3 ± 17.2	
No drink	2658 (67.9)	2453 (67.7)	205 (70.2)		69.3 ± 17.6	
Eating behavior **				<0.001		0.642
Eat less healthy or unchanged	2931(74.6)	2686 (73.9)	245 (83.6)		69.6 ± 17.8	
Eat healthier	996 (25.4)	948 (26.1)	48 (16.4)		69.9 ± 16.4	
Physical activity, MET-min/wk				<0.001		<0.001
Tertile 1 (0.0–748.5)	1314 (33.3)	1159 (31.7)	155 (52.7)		65.3 ± 19.1	
Tertile 2 (≥748.5–3399.0)	1316 (33.3)	1244 (34.1)	72 (24.5)		70.7 ± 16.1	
Tertile 3 (≥3399.0–4453.8)	1317 (33.4)	1250 (34.2)	67 (22.8)		72.7 ± 16.2	
HL index, mean ± SD	29.9 ± 7.7	30.4 ± 7.5	24.5 ± 8.4	<0.001		
HRQoL score, mean ± SD	69.6 ± 17.5	71.3 ± 16.5	48.1 ± 14.5	<0.001		

Abbreviations: PHQ, patient health questionnaire; HRQoL, health-related quality of life; SD, standard deviation; OPD, outpatient department; S-COVID-19-S, suspected corona virus disease-2019 symptoms; BMI, body mass index; MET-min/week, metabolic equivalent task scored in minute per week; HL, health literacy. ^1^ Result of Chi-square test. ^2^ Result of ANOVA test. * The suspected COVID-19 symptoms including common symptom (fever, cough, dyspnea), less common symptom (myalgia, fatigue, sputum production, confusion, headache, sore throat, rhinorrhea, chest pain, hemoptysis, diarrhea, and nausea/vomiting). ** People were asked whether their eating behavior is getting worse, better, or unchanged during COVID-19 outbreak as compared to those before the outbreak.

**Table 2:** Determinants of depression and health-related quality of life among people visiting outpatient department (*n* = 3947).

	Depression (PHQ ≥ 10)				HRQoL			
	Bivariate		Multivariate		Bivariate		Multivariate	
	OR (95%CI)	*p*	OR (95%CI)	*p*	B (95%CI)	*p*	B (95%CI)	*p*
Reasons to visit OPD *								
Without S-COVID-19-S	1.00		1.00		0.00		0.00	
With S-COVID-19-S	3.69 (2.88, 4.73)	<0.001	2.88 (2.18, 3.80)	<0.001	−11.53 (−12.61, −10.45)	<0.001	−7.92 (−8.95, −6.89)	<0.001
Age, year, mean ± SD								
18–39	1.00		1.00		0.00		0.00	
40–59	1.10 (0.80, 1.50)	0.554	0.81 (0.56, 1.15)	0.244	−4.71 (−5.91, −3.50)	<0.001	−0.91 (−2.11, 0.30)	0.141
≥60	2.69 (2.04, 3.55)	<0.001	1.15 (0.79, 1.68)	0.464	−13.65 (−14.96, −12.33)	<0.001	−3.60 (−5.13, −2.08)	<0.001
Gender								
Women	1.00		1.00		0.00		0.00	
Men	0.85 (0.66, 1.08)	0.171	0.92 (0.71, 1.20)	0.556	2.85 (1.76, 3.95)	<0.001	1.89 (0.82, 2.95)	0.001
Marital status								
Never married	1.00				0.00		0.00	
Ever married	0.99 (0.75, 1.32)	0.957			−7.31 (−8.61, −6.02)	<0.001	−2.82 (−4.12, −1.51)	<0.001
Education								
Elementary school or Illiterate	1.00		1.00		0.00		0.00	
Junior high school	0.33 (0.22, 0.50)	<0.001	0.72 (0.45, 1.16)	0.175	13.20 (11.11, 15.28)	<0.001	6.82 (4.85, 8.78)	< 0.001
Senior high school	0.34 (0.23, 0.50)	<0.001	1.24 (0.78, 1.99)	0.363	17.15 (15.12, 19.17)	<0.001	6.70 (4.71, 8.69)	<0.001
College/university or higher	0.48 (0.34, 0.67)	<0.001	2.12 (1.34, 3.35)	0.001	16.40 (14.45, 18.35)	<0.001	4.70 (2.66, 6.75)	<0.001
Occupation								
Employed	1.00				0.00		0.00	
Own business	0.92 (0.65, 1.30)	0.627			−2.88 (−4.42, −1.34)	<0.001	2.25 (0.73, 3.77)	0.004
Others	1.21 (0.88, 1.68)	0.243			−4.53 (−6.01, −3.05)	<0.001	0.18 (−1.26, 1.61)	0.808
Ability to pay for medication								
Very or fairly difficult	1.00		1.00		0.00		0.00	
Very or fairly easy	0.52 (0.41, 0.67)	<0.001	1.02 (0.77, 1.34)	0.909	8.13 (7.06, 9.19)	<0.001	2.75 (1.74, 3.76)	<0.001
Social status								
Low	1.00		1.00		0.00		0.00	
Middle or high	0.29 (0.22, 0.39)	<0.001	0.45 (0.33, 0.63)	<0.001	11.54 (9.92, 13.17)	<0.001	4.62 (3.09, 6.15)	<0.001
BMI, kg/m^2^								
Normal weight (BMI < 25.0)	1.00		1.00		0.00			
Overweight/obese (BMI ≥ 25.0)	0.68 (0.44, 1.06)	0.087	0.90 (0.57, 1.44)	0.669	1.05 (−0.71, 2.80)	0.241		
Comorbidity								
None	1.00		1.00		0.00		0.00	
One or more	1.39 (1.02, 1.87)	0.034	0.98 (0.69, 1.39)	0.907	−7.44 (−8.93, −5.96)	<0.001	−2.81 (−4.18, −1.45)	<0.001
Smoking								
Smoke	1.00				0.00			
No smoke	0.91 (0.64, 1.29)	0.583			−0.38 (−2.06, 1.31)	0.662		
Drinking alcohol								
Drink	1.00				0.00		0.00	
No drink	1.12 (0.87, 1.46)	0.379			−1.05 (−2.22, 0.13)	0.080	1.74 (0.61, 2.87)	0.003
Eating behavior *								
Eat less healthy or unchanged	1.00		1.00		0.00			
Eat healthier	0.56 (0.40, 0.76)	<0.001	0.59 (0.42, 0.83)	0.003	0.30 (−0.96, 1.55)	0.642		
Physical activity, MET-min/wk								
Tertile 1 (0.0–748.5)	1.00		1.00		0.00		0.00	
Tertile 2 (≥748.5–3399.0)	0.43 (0.32, 0.58)	<0.001	0.59 (0.42, 0.82)	0.001	5.44 (4.12, 6.75)	<0.001	1.11 (−0.08, 2.29)	0.067
Tertile 3 (≥3399.0–44538)	0.40 (0.30, 0.54)	<0.001	0.56 (0.40, 0.78)	0.001	7.37 (6.05, 8.68)	<0.001	2.72 (1.52, 3.92)	<0.001
HL index (1 score increment)	0.91 (0.90, 0.93)	<0.001	0.93 (0.91, 0.95)	<0.001	0.94 (0.87, 1.00)	<0.001	0.59 (0.52, 0.66)	<0.001

Abbreviations: PHQ, patient health questionnaire; HRQoL, health-related quality of life; OR, odd ratio; B, regression coefficient; CI, confidence interval; OPD, outpatient department; S-COVID-19-S, suspected corona virus disease-2019 symptoms; BMI, body mass index; MET-min/week, metabolic equivalent task scored in minute per week; HL, health literacy. * People were asked whether their eating behavior is getting worse, better, or unchanged during Covid-19 outbreak as compared to those before the outbreak.

**Table 3:** Interaction effect of suspected Covid-19 symptoms and health literacy on depression and health-related quality of life (*n* = 3947).

Interaction	Depression (PHQ ≥ 10)				HRQoL			
	Model 1		Model 2		Model 1		Model 2	
	OR (95%CI)	*p*	OR (95%CI)	*p*	B (95%CI)	*p*	B (95%CI)	*p*
Without S-COVID-19-S	1.00		1.00		0.00		0.00	
With S-COVID-19-S	12.09 (5.06, 28.89)	<0.001	9.70 (4.02, 23.41)	<0.001	−24.88 (−28.86, −20.90)	<0.001	−20.62 (−24.63, −16.62)	<0.001
HL index (1-score increment)	0.95 (0.93, 0.97)	<0.001	0.95 (0.93, 0.97)	<0.001	0.66 (0.58, 0.73)	<0.001	0.45 (0.37, 0.54)	<0.001
With S-COVID-19-S × HL index (1-score increment)	0.95 (0.92, 0.98)	0.001	0.96 (0.93, 0.99)	0.004	0.54 (0.41, 0.68)	<0.001	0.43 (0.30, 0.57)	<0.001

Abbreviations: PHQ, patient health questionnaire; HRQoL, health-related quality of life; OR, odd ratio; B, regression coefficient; CI, confidence interval; S-COVID-19-S, suspected corona virus disease-2019 symptoms; HL, health literacy. Model 1: interaction between S-COVID-19-S and health literacy, Model 2: interaction between S-COVID-19-S and health literacy after adjusted for education, social status, eating behavior, and physical activity, as analyzed for depression; adjusted for age, gender, marital status, education, occupation, ability to pay for medication, social status, comorbidity, drinking and physical activity as analyzed for HRQoL.

## Supplementary Materials

The following are available online at https://www.mdpi.com/2077-0383/9/4/965/s1, Table S1: Interaction effect of common suspected Covid-19 symptoms and health literacy on depression and health-related quality of life.

## References

[1] Ren L.-L., Wang Y.-M., Wu Z.-Q., Xiang Z.-C., Guo L., Xu T., Jiang Y.-Z., Xiong Y., Li Y.-J., Li X.-W. (2020). Identification of a novel coronavirus causing severe pneumonia in human: A descriptive study. Chin. Med. J. (Engl.).

[2] Wang C., Horby P.W., Hayden F.G., Gao G.F. (2020). A novel coronavirus outbreak of global health concern. Lancet.

[3] Thompson R. (2020). Pandemic potential of 2019-nCoV. Lancet Infect. Dis..

[4] Wu J.T., Leung K., Leung G.M. (2020). Nowcasting and forecasting the potential domestic and international spread of the 2019-nCoV outbreak originating in Wuhan, China: A modelling study. Lancet.

[5] World Health Organization (WHO) Statement on the Second Meeting of the International Health Regulations (2005) Emergency Committee Regarding the Outbreak of Novel Coronavirus (2019-nCoV).

[6] World Health Organisation Coronavirus Disease (COVID-2019) Situation Reports.

[7] Ministry of Health Coronavirus Disease (COVID-19) Outbreak in Vietnam. https://ncov.moh.gov.vn/.

[8] World Health Organisation WHO Director-General’s Opening Remarks at the Media Briefing on COVID-19–11 March 2020.

[9] World Health Organisation Coronavirus Disease (COVID-19) Outbreak.

[10] World Health Organization (WHO) Home Care for Patients with Suspected Novel Coronavirus (nCoV) Infection Presenting with Mild Symptoms and Management of Contacts: Interim Guidance.

[11] World Health Organization (WHO) Clinical Management of Severe Acute Respiratory Infection When Novel Coronavirus (nCoV) Infection is Suspected: Interim Guidance.

[12] World Health Organization (WHO) Infection Prevention and Control during Health Care When Novel Coronavirus (nCoV) Infection Is Suspected: Interim Guidance.

[13] Zhou T., Liu Q., Yang Z., Liao J., Yang K., Bai W., Lu X., Zhang W. (2020). Preliminary prediction of the basic reproduction number of the Wuhan novel coronavirus 2019-nCoV. J. Evid. Based Med..

[14] World Health Organization WHO Director-General’s Statement on IHR Emergency Committee on Novel Coronavirus (2019-nCoV).

[15] Thompson R.N. (2020). Novel Coronavirus Outbreak in Wuhan, China, 2020: Intense Surveillance Is Vital for Preventing Sustained Transmission in New Locations. J. Clin. Med..

[16] Deng S.-Q., Peng H.-J. (2020). Characteristics of and Public Health Responses to the Coronavirus Disease 2019 Outbreak in China. J. Clin. Med..

[17] Bao Y., Sun Y., Meng S., Shi J., Lu L. (2020). 2019-nCoV epidemic: Address mental health care to empower society. Lancet.

[18] Xu Z., Li S., Tian S., Li H., Kong L.-Q. (2020). Full spectrum of COVID-19 severity still being depicted. Lancet.

[19] Jack A. (2015). Why the panic? South Korea’s MERS response questioned. BMJ (Clin. Res. Ed.).

[20] Abdel-Moneim A.S. (2015). Middle-East respiratory syndrome coronavirus: Is it worth a world panic?. World J. Virol..

[21] Shimizu K. (2020). 2019-nCoV, fake news, and racism. Lancet.

[22] Brooks S.K., Webster R.K., Smith L.E., Woodland L., Wessely S., Greenberg N., Rubin G.J. (2020). The psychological impact of quarantine and how to reduce it: Rapid review of the evidence. Lancet.

[23] Lancet (2009). The health illiteracy problem in the USA. Lancet.

[24] Sørensen K., Van den Broucke S., Brand H., Fullam J., Doyle G., Pelikan J., Slonszka Z. (2012). Health literacy and public health: A systematic review and integration of definitions and models. BMC Public Health.

[25] Watson R. (2011). Europeans with poor “health literacy” are heavy users of health services. BMJ.

[26] Greenhalgh T. (2015). Health literacy: Towards system level solutions. BMJ.

[27] Ishikawa H., Yano E. (2008). Patient health literacy and participation in the healthcare process. Health Expect..

[28] The World Bank Country Profile: Vietnam.

[29] Duong T.V., Aringazina A., Baisunova G., Pham T.V., Pham K.M., Truong T.Q., Nguyen K.T., Oo W.M., Mohamad E., Su T.T. (2017). Measuring health literacy in Asia: Validation of the HLS-EU-Q47 survey tool in six Asian countries. J. Epidemiol..

[30] Duong T.V., Aringazina A., Baisunova G., Nurjanah N., Pham T.V., Pham K.M., Truong T.Q., Nguyen K.T., Oo W.M., Su T.T. (2019). Development and validation of a new short-form health literacy instrument (HLS-SF12) for the general public in six Asian countries. Health Lit. Res. Pract..

[31] Rudd R.E. (2007). Health literacy skills of US adults. Am. J. Health Behav..

[32] Mackert M. (2015). Introduction to a Colloquium: Challenges and Opportunities in Advancing Health Literacy Research. Health Commun..

[33] Qin L., Xu H. (2016). A cross-sectional study of the effect of health literacy on diabetes prevention and control among elderly individuals with prediabetes in rural China. BMJ Open.

[34] Raynor D.K. (2012). Health literacy. BMJ.

[35] Hu Z., Qin L., Xu H. (2019). Association between diabetes-specific health literacy and health-related quality of life among elderly individuals with pre-diabetes in rural Hunan Province, China: A cross-sectional study. BMJ Open.

[36] Kugbey N., Meyer-Weitz A., Oppong Asante K. (2019). Access to health information, health literacy and health-related quality of life among women living with breast cancer: Depression and anxiety as mediators. Patient Educ. Couns..

[37] Kirk J.K., Grzywacz J.G., Arcury T.A., Ip E.H., Nguyen H.T., Bell R.A., Saldana S., Quandt S.A. (2012). Performance of health literacy tests among older adults with diabetes. J. Gen. Intern. Med..

[38] Editorial Team Overview of Novel Coronavirus (2019-nCoV). BMJ Best Practice. https://bestpractice.bmj.com/topics/en-gb/3000165.

[39] Craig C.L., Marshall A.L., Sjöström M., Bauman A.E., Booth M.L., Ainsworth B.E., Pratt M., Ekelund U., Yngve A., Sallis J.F. (2003). International physical activity questionnaire: 12-country reliability and validity. Med. Sci. Sports Exerc..

[40] Pham T., Bui L., Nguyen A., Nguyen B., Tran P., Vu P., Dang L. (2019). The prevalence of depression and associated risk factors among medical students: An untold story in Vietnam. PLoS ONE.

[41] Tran D.V., Lee A.H., Au T.B., Nguyen C.T., Hoang D.V. (2013). Reliability and validity of the International Physical Activity Questionnaire-Short Form for older adults in Vietnam. Health Promot. J. Aust..

[42] Lee P.H., Macfarlane D.J., Lam T.H., Stewart S.M. (2011). Validity of the international physical activity questionnaire short form (IPAQ-SF): A systematic review. Int. J. Behav. Nutr. Phys. Act..

[43] Duong T.V., Nguyen T.T.P., Pham K.M., Nguyen K.T., Giap M.H., Tran T.D.X., Nguyen C.X., Yang S.-H., Su C.-T. (2019). Validation of the Short-Form Health Literacy Questionnaire (HLS-SF12) and Its Determinants among People Living in Rural Areas in Vietnam. Int. J. Environ. Res. Public Health.

[44] HLS-EU Consortium Comparative Report of Health Literacy in Eight EU Member States. The European Health Literacy Project 2009–2012.

[45] Kroenke K., Spitzer R.L., Williams J.B.W. (2001). The PHQ-9: Validity of a brief depression severity measure. J. Gen. Intern. Med..

[46] Nguyen T.Q., Bandeen-Roche K., Bass J.K., German D., Nguyen N.T.T., Knowlton A.R. (2016). A tool for sexual minority mental health research: The Patient Health Questionnaire (PHQ-9) as a depressive symptom severity measure for sexual minority women in Viet Nam. J. Gay Lesbian Ment. Health.

[47] Manea L., Gilbody S., McMillan D. (2015). A diagnostic meta-analysis of the Patient Health Questionnaire-9 (PHQ-9) algorithm scoring method as a screen for depression. Gen. Hosp. Psychiatry.

[48] Levis B., Benedetti A., Thombs B.D. (2019). Accuracy of Patient Health Questionnaire-9 (PHQ-9) for screening to detect major depression: Individual participant data meta-analysis. BMJ.

[49] Hays R.D., Morales L.S. (2001). The RAND-36 measure of health-related quality of life. Ann. Med..

[50] Ngo-Metzger Q., Sorkin D.H., Mangione C.M., Gandek B., Hays R.D. (2008). Evaluating the SF-36 Health Survey (Version 2) in Older Vietnamese Americans. J. Aging Health.

[51] Hays R.D., Kallich J., Mapes D., Coons S., Amin N., Carter W.B., Kamberg C. (1997). Kidney Disease Quality of Life Short Form (KDQOL-SF), Version 1.3: A Manual for Use and Scoring.

[52] Hays R.D., Sherbourne C.D., Mazel R.M. (1993). The RAND 36-Item Health Survey 1.0. Health Econ..

[53] National Center for Immunization and Respiratory Diseases (NCIRD) What Healthcare Personnel Should Know about Caring for Patients with Confirmed or Possible 2019-nCoV Infection.

[54] World Health Organization (WHO) Novel Coronavirus (2019-nCoV) Technical Guidance.

[55] Maldonado G., Greenland S. (1993). Simulation Study of Confounder-Selection Strategies. Am. J. Epidemiol..

[56] IBM SPSS (2011). IBM SPSS Statistics for Windows, Version 20.0.

[57] Gostin L.O., Hodge J.G. (2020). US Emergency Legal Responses to Novel Coronavirus: Balancing Public Health and Civil Liberties. JAMA.

[58] Wang C.J., Ng C.Y., Brook R.H. (2020). Response to COVID-19 in Taiwan: Big Data Analytics, New Technology, and Proactive Testing. JAMA.

[59] Chen J. (2020). Pathogenicity and Transmissibility of 2019-nCoV-A Quick Overview and Comparison with Other Emerging Viruses. Microbes Infect..

[60] The Government of the Socialist Republic of Vietnam Government in Action. Hanoi, Vietnam, Online Newspaper of the Government. http://news.chinhphu.vn/Home/Government-in-action.vgp.

[61] Smith N., Fraser M. (2020). Straining the System: Novel Coronavirus (COVID-19) and Preparedness for Concomitant Disasters. Am. J. Public Health.

[62] Adalja A.A., Toner E., Inglesby T.V. (2020). Priorities for the US Health Community Responding to COVID-19. JAMA.

[63] Duong T.V., Sørensen K., Pelikan J., Van den Broucke S., Lin I.F., Lin Y.-C., Huang H.-L., Chang P.W. (2017). Health-related behaviors moderate the association between age and self-reported health literacy among Taiwanese women. Women Health.

[64] Sun X., Yang S., Fisher E.B., Shi Y., Wang Y., Zeng Q., Ji Y., Chang C., Du W. (2014). Relationships of Health Literacy, Health Behavior, and Health Status Regarding Infectious Respiratory Diseases: Application of a Skill-Based Measure. J. Health Commun..

[65] Castro-Sánchez E., Chang P.W.S., Vila-Candel R., Escobedo A.A., Holmes A.H. (2016). Health literacy and Infectious Diseases: Why does it matter?. Int. J. Infect. Dis..

[66] Huong N.T., Ha L.T.H., Tien T.Q. (2017). Determinants of Health-Related Quality of Life Among Elderly: Evidence From Chi Linh Town, Vietnam. Asia Pac. J. Public Health.

[67] Hoi L.V., Chuc N.T., Lindholm L. (2010). Health-related quality of life, and its determinants, among older people in rural Vietnam. BMC Public Health.

[68] Ha N.T., Duy H.T., Le N.H., Khanal V., Moorin R. (2014). Quality of life among people living with hypertension in a rural Vietnam community. BMC Public Health.

[69] Pham T., Nguyen N.T.T., ChieuTo S.B., Pham T.L., Nguyen T.X., Nguyen H.T.T., Nguyen T.N., Nguyen T.H.T., Nguyen Q.N., Tran B.X. (2018). Sex Differences in Quality of Life and Health Services Utilization among Elderly People in Rural Vietnam. Int. J. Environ. Res. Public Health.

[70] Nguyen H.V., Tran T.T., Nguyen C.T., Tran T.H., Tran B.X., Latkin C.A., Ho C.S.H., Ho R.C.M. (2019). Impact of Comorbid Chronic Conditions to Quality of Life among Elderly Patients with Diabetes Mellitus in Vietnam. Int. J. Environ. Res. Public Health.

[71] Nguyen L.H., Tran B.X., Hoang Le Q.N., Tran T.T., Latkin C.A. (2017). Quality of life profile of general Vietnamese population using EQ-5D-5L. Health Qual. Life Outcomes.

[72] Ngo C.Q., Phan P.T., Vu G.V., Pham Q.L.T., Nguyen L.H., Vu G.T., Tran T.T., Nguyen H.L.T., Tran B.X., Latkin C.A. (2019). Effects of Different Comorbidities on Health-Related Quality of Life among Respiratory Patients in Vietnam. J. Clin. Med..

[73] Ministry of Education and Training Postpone the New Semester to Prevent Covid-19 Outbreak in 63/63 Cities and Provinces. https://moet.gov.vn/tintuc/Pages/phong-chong-nCoV.aspx?ItemID=6454.

[74] Mammen G., Faulkner G. (2013). Physical activity and the prevention of depression: A systematic review of prospective studies. Am. J. Prev. Med..

[75] Kvam S., Kleppe C.L., Nordhus I.H., Hovland A. (2016). Exercise as a treatment for depression: A meta-analysis. J. Affect. Disord..

[76] Santos M.V.F.D., Campos M.R., Fortes S.L.C.L. (2019). Relationship of alcohol consumption and mental disorders common with the quality of life of patients in primary health care. Cien. Saude Colet..

[77] Daeppen J.-B., Faouzi M., Sanchez N., Rahhali N., Bineau S., Bertholet N. (2014). Quality of life depends on the drinking pattern in alcohol-dependent patients. Alcohol Alcohol..

[78] Levola J., Aalto M., Holopainen A., Cieza A., Pitkänen T. (2014). Health-related quality of life in alcohol dependence: A systematic literature review with a specific focus on the role of depression and other psychopathology. Nord. J. Psychiatry.

[79] Molendijk M., Molero P., Ortuño Sánchez-Pedreño F., Van der Does W., Angel Martínez-González M. (2018). Diet quality and depression risk: A systematic review and dose-response meta-analysis of prospective studies. J. Affect. Disord..

[80] Li Y., Lv M.-R., Wei Y.-J., Sun L., Zhang J.-X., Zhang H.-G., Li B. (2017). Dietary patterns and depression risk: A meta-analysis. Psychiatry Res..

[81] Opie R.S., Itsiopoulos C., Parletta N., Sanchez-Villegas A., Akbaraly T.N., Ruusunen A., Jacka F.N. (2017). Dietary recommendations for the prevention of depression. Nutr. Neurosci..

[82] Martínez-González M.A., Sánchez-Villegas A. (2016). Food patterns and the prevention of depression. Proc. Nutr. Soc..

