# Supplementary material for: People with Suspected COVID-19 Symptoms Were More Likely Depressed and Had Lower Health-Related Quality of Life: The Potential Benefit of Health Literacy
Source: J Clin Med. 2020 Mar 31;9(4):965. doi: 10.3390/jcm9040965 (PMC7231234; doi:10.3390/jcm9040965)
Supplement: Supplementary file 1 [file jcm-09-00965-s001.pdf]

Table S1. Interaction effect of common suspected Covid-19 symptoms and health literacy on depression and health-related quality of life (*n*=3247).

**Interaction**	**Depression (PHQ ≥ 10)**				**HRQoL**			
	**Model 1**		**Model 2**		**Model 1**		**Model 2**	
	**OR (95%CI)**	***p***	**OR (95%CI)**	***p***	**B (95%CI)**	***p***	**B (95%CI)**	***p***
Without S-Covid-19-S	1.00		1.00		0.00		0.00	
With common S-Covid-19-S	13.54 (4.90, 37.39)	<0.001	10.43 (3.68, 29.53)	<0.001	-30.52 (-35.55, -25.50)	<0.001	-27.10 (-32.21, -21.99)	<0.001
HL index (1-score increment)	0.95 (0.93, 0.97)	<0.001	0.95 (0.93, 0.98)	<0.001	0.66 (0.58, 0.73)	<0.001	0.44 (0.36, 0.52)	<0.001
With common S-Covid-19-S × HL index (1-score increment)	0.95 (0.92, 0.99)	0.009	0.96 (0.93, 1.00)	0.038	0.70 (0.52, 0.87)	<0.001	0.63 (0.45, 0.80)	<0.001

Abbreviations: PHQ, patient health questionnaire; HRQoL, health-related quality of life; OR, odd ratio; B, regression coefficient; CI, confidence interval; S-Covid-19-S, suspected coronavirus disease-2019 symptoms; HL, health literacy.

Model 1: interaction between common S-Covid-19-S and health literacy

Model 2: interaction between common S-Covid-19-S and health literacy after adjusted for education, social status, eating behavior, and physical activity, as analyzed for depression; adjusted for age, gender, marital status, education, occupation, ability to pay for medication, social status, comorbidity, drinking and physical activity as analyzed for HRQoL.
